# Dorsal hippocampal CA1 NMDA receptors mediate the interactive effects of quetiapine and lithium on memory retention in male rats

**DOI:** 10.22038/IJBMS.2023.69714.15177

**Published:** 2023

**Authors:** Majid Jafari-Sabet, Shiva Amiri, Sahar Emami, Helia Aghamiri, Navid Fatahi, Fariborz Keyhanfar

**Affiliations:** 1 Department of Pharmacology, School of Medicine, Iran University of Medical Sciences, Tehran, Iran; 2 Razi Drug Research Center, School of Medicine, Iran University of Medical Sciences, Tehran, Iran; 3 Pharmaceutics Research Center, Institute of Neuropharmacology, Kerman University of Medical Sciences, Kerman, Iran; 4 Department of Toxicology and Pharmacology, Faculty of Pharmacy, Kerman University of Medical Sciences, Kerman, Iran

**Keywords:** Dorsal hippocampus, Lithium, MK-801, N-methyl-D-aspartic acid, Quetiapine

## Abstract

**Objective(s):**

Lithium and quetiapine are administered simultaneously as a treatment for bipolar disorder. The concurrent use of these two drugs has been observed to affect the neurobiological mechanisms underlying learning and memory. To clarify the precise mechanisms involved, we evaluated the possible role of the dorsal hippocampal CA1 NMDA receptors in the interactive effects of lithium and quetiapine in memory consolidation.

**Materials and Methods:**

The dorsal hippocampal CA1 regions of adult male Wistar rats were bilaterally cannulated, and a single-trial step-through inhibitory avoidance apparatus was used to assess memory consolidation.

**Results:**

Post-training administration of certain doses of lithium (20, 30, and 40 mg/kg, IP) diminished memory consolidation. Post-training administration of higher doses of quetiapine (5, 10, and 20 mg/kg, IP) augmented memory consolidation. Post-training administration of certain doses of quetiapine (2.5, 5, 10, and 20 mg/kg) dose-dependently restored lithium-induced memory impairment. Post-training microinjection of ineffective doses of the NMDA (10^-5^ and 10^-4 ^µg/rat, intra-CA1) plus an ineffective dose of quetiapine (2.5 mg/kg) restored the lithium-induced memory impairment. Post-training microinjection of ineffective doses of the noncompetitive NMDA receptor antagonist, MK-801 (0.0625 and 0.0125 μg/rat, intra-CA1), diminished the quetiapine-induced (10 mg/kg) memory improvement in lithium-induced memory impairment.

**Conclusion:**

These findings suggest a functional interaction between lithium and quetiapine through hippocampal CA1 NMDA receptor mechanisms in memory consolidation.

## Introduction

Bipolar disorder (BPD) is a chronic, debilitating, and recurrent disorder that can seriously affect the day-to-day tasks of patients (1, 2). Cognitive dysfunction such as short- and long-term memory deficits are continuously reported across BPD periods (3). On the other hand, drug-related cognitive deficits such as memory dysfunction are a major problem in psychotic patients treated with antipsychotic drugs (4). 

Lithium is a mood stabilizer recognized as the first line of treatment for BPD, and strong evidence has shown its effectiveness in preventing relapse and re-hospitalization and reducing suicidality. (5-7). Multiple clinical studies have indicated that being treated with lithium negatively affects several cognitive functions, learning, and memory processes in the average population and bipolar patients (8-10). Furthermore, experiments investigating the role of lithium in learning and memory processes have found that administration of lithium impairs memory in various hippocampus-dependent memory tasks (11, 12). It has been demonstrated that lithium can modulate the function of N-methyl-D-aspartic acid (NMDA) as a partial agonist of glutamate ionotropic receptors and also regulate signal transduction pathways in many brain regions, such as the hippocampus (11-13). 

Quetiapine is an atypical antipsychotic drug and is clinically used to treat certain mental-mood states such as schizophrenia, BPD, and abrupt episodes of mania or depression associated with BPD (14-16).

It has been reported that quetiapine and other atypical antipsychotic drugs induce neuronal plasticity and synaptic remodeling in multiple brain regions such as the striatum, prefrontal cortex, and hippocampus (17). Furthermore, it has been indicated that quetiapine positively impacts cognitive functions such as spatial memory, verbal working memory, reasoning, problem-solving, and verbal fluency (18, 19). It has been revealed that systemic administration of quetiapine increases the extracellular levels of multiple neurotransmitters such as norepinephrine, dopamine, and glutamate in some brain regions (20, 21). It has been well established that the dorsal hippocampal CA1 region is essential to forming memories about experienced single-trial inhibitory avoidance tasks (11, 22-25). Furthermore, a growing body of evidence has indicated that CA1 NMDA receptor-dependent plasticity is essential for memory formation (11, 26, 27). 

Given that lithium and quetiapine are widely used in acute and maintenance treatments for BPD (28), the combination of these agents provides superior efficacy (16, 29). As CA1 NMDA receptors play a crucial role in memory consolidation and retrieval, synaptic plasticity, and memory formation, the main aims of the present study were to identify the effects of single-dose intraperitoneal (IP) administration of lithium and/or quetiapine on memory consolidation and the effects of intra-CA1 microinjection of NMDA and/or MK-801 on the impact of lithium and quetiapine on memory consolidation in step-through inhibitory avoidance tasks in rats.

## Materials and Methods

***Animals***

Male Wistar rats (Iran University of Medical Sciences, Tehran, Iran), weighing 200–220 g, at the time of the experiments, were used. The animals were accommodated 4 per cage in a room and were maintained under a 12-hr light/12-hr dark cycle (lights on at 07:00 am) and with a controlled temperature (22 ± 2 °C). All animals were allowed to adjust to the laboratory setting for at least one week before the experiment and were handled for 5 min/day during this habituation period. They had free access to water and food at all times except during the training and testing phases. During the light phase between 09:00 am and 3:00 pm, training and testing were conducted in a quiet environment.

All animal experiments were conducted in conformity with the UK Animals (Scientific Procedures) Act 1986 and the associated guidelines and EU Directive 2010/63/EU for animal experiments, and they were approved by the Ethics Committee of Iran University of Medical Sciences (ethics code: IR.IUMS.FMD.REC.1398.447).

***Drugs***

 The drugs used in the present investigation were lithium chloride (Merck, Germany), quetiapine, N-methyl-D-aspartate (NMDA), and (5S, 10R)-(+)-5-methyl-10, 11-dihydro-5Hibenzo [a,d] cycloheptan-5,10-imine maleate ((+)-MK-801 maleate) (Tocris, Bristol, UK). Quetiapine was dissolved in sterile 0.9 % saline and a drop of glacial acetic acid. Lithium, NMDA, and MK-801 were dissolved in sterile 0.9% saline. Control groups received either saline or a suitable vehicle (one drop of glacial acetic acid in sterile 0.9% saline). Quetiapine and lithium were administered intraperitoneally (IP), 1 ml/kg. NMDA and MK-801 were bilaterally microinjected into the dorsal hippocampal CA1 regions (intra-CA1) at a volume of 1 μl/rat (0.5 μl per side). All drugs were prepared just prior to the experiments.

Considering that locomotor activity may affect the measurement of memory formation,

Due to the possibility that changes in locomotor activity may affect memory formation, the doses of drugs were selected based on previous studies (11, 22, 30), pilot experiments (using the open field apparatus), and some other studies (31-33). No significant effect has been observed on locomotor activity after administration of these doses.

***Inhibitory avoidance apparatus***

In order to evaluate memory consolidation, animals were trained and tested in a single-trial step-through inhibitory avoidance apparatus (Borj Sanat Company, Tehran, Iran) (11, 22). Briefly, the task consisted of an opaque Plexiglas box consisting of two equally sized white and black compartments (20 cm × 20 cm × 40 cm) separated by a guillotine-like door (8 cm×8 cm). The floor of the black compartment was constructed with stainless-steel bars (0.5 cm in diameter and spaced 1 cm apart). A stimulator isolated from the grid floor of the black compartment delivered intermittent electric shocks (Frequency: 50 Hz, electrical current: 1.5-mA, duration: 5 sec).

***Behavioral procedure and data collection***

***Training and testing phase***

To evaluate memory consolidation, the same protocol as the previous study was used (11). In this one-trial learning task, the animals were allowed to adapt to the experiment room for 60 min before the training or testing sessions during the light phase of the cycle. In the training trial, each animal was gently placed in the white compartment for 10 sec, after which the guillotine door was opened, and then the time the animal waited before crossing to the black compartment was recorded as latency. If each animal delayed more than 120 sec to cross to the other side it was excluded from the experiment. As soon as the animal entered with all four paws to the next (black) compartment, the door was closed and a foot shock (1.5-mA, 5 sec) was immediately delivered to the metal grid floor by an isolated stimulator (Borj Sanat Co., Tehran, Iran). The animal was then removed from the apparatus, and the drugs immediately were administered post-training (after training) intraperitoneally (IP) and/or intra-dorsal hippocampal (intra-CA1). 

To evaluate memory consolidation, a retention test was performed twenty-four hours after training. Each animal was placed in the white compartment and after 10 sec the guillotine door was opened. The step-through latency for entering into the black (shock) compartment was measured as a measure of memory consolidation. It should be noted that during test sessions, no electric shock was delivered to the animals. 

The test session ended when the animal entered the black compartment or stayed in the white compartment for 300 sec. An upper cut-off time of 300 sec was set. All experiments were carried out between 9:00 AM and 3:00 PM.

***Surgical and cannula guide implantation***

 The rats were anesthetized using intraperitoneal injections of ketamine hydrochloride (50 mg/kg) plus xylazine (4 mg/kg) and then located in a stereotaxic device (11). Following skin cutting and cleaning of the skull, two 22-gauge stainless-steel guide cannulas were implanted (bilaterally) 1 mm above the intended site of infusion, according to the atlas of Paxinos and Watson (34). Stereotaxic coordinates for the CA1 regions of the dorsal hippocampus were −3.3 mm posterior to bregma, −2 mm lateral to the sagittal suture, and −2 ventral to the dorsal surface of the skull (depending on body weight). An electric drill with a 1 mm bit was used to drill a small hole in the anterolateral skull to the target insertion site, and two screws (1 mm diameter) were inserted into the skull to serve as anchors for the dental cement. After making sure the skull was completely dry, liquid dental cement was applied completely around the screw and cannula to secure both in place. Stainless steel stylets (27-gauge) were incorporated into the guide cannula to hinder clogging. All animals were permitted to recover from surgery and anesthesia for one week.

***Intra-CA1 injection procedures***

Specimen preparation was conducted as in our previous report (11) as follows: 

For intra-CA1 infusion of the drugs, the animals were mildly harnessed by hand, and the stylets were picked up from the guide cannula and replaced by 27-gauge injection needles (1 mm under the tip of the guide cannula). Each microinjection unit was attached to a 1-µl Hamilton syringe using polyethylene tubing. The left and right CA1 were infused with 0.5 μl solution on each side (1 μl/rat) over a 60-sec period. Injection needles were left in site for an additional 60 sec to make sure of drug infusion and afterward, the stylets were reinserted into the guide cannulas.

***Experimental design***

**Figure 1:**
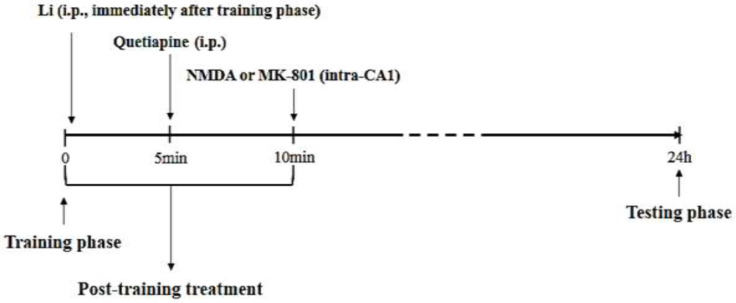
A schematic diagram of drug administration in male Wistar rats

Eight male Wistar rats were used in each experimental group. In experiments where the animals received one or two injections, the control groups also received one or two saline or vehicle injections (Figure 1).

***Experiment 1***

This experiment investigated the effects of post-training IP injection of different doses of lithium on memory consolidation. Five groups of animals were used. The control group received saline (1 ml/kg, IP) immediately after training (post-training). Another four groups of animals received post-training administration of lithium (10, 20, 30, and 40 mg/kg, IP).

***Experiment 2***

This experiment investigated the effects of post-training IP injection of different doses of quetiapine on memory consolidation. Seven groups of animals were used. The control groups received saline and/or the vehicle (1 ml/kg, IP) immediately after training (post-training). Another five groups of animals received post-training administration of quetiapine (1.25, 2.5, 5, 10, and 20 mg/kg, IP).

***Experiment 3***

 This experiment investigated the effects of post-training IP injection of certain doses of quetiapine on lithium-induced impairment of memory consolidation.

Seven groups of animals were used. The control group received post-training administration of saline (1 ml/kg, IP). Another six groups of animals received post-training administration of saline and/or different doses of quetiapine (1.25, 2.5, 5, 10, and 20 mg/kg, IP) plus lithium (40 mg/kg, IP) with 5-min intervals.

***Experiment 4***

This experiment investigated the effects of post-training intra-CA1 microinjection of specific doses of NMDA (an NMDA receptor agonist) on memory improvement induced by quetiapine on the lithium-induced memory consolidation impairment. 

Seven groups of animals were used. The control group received post-training microinjection of saline (1 μl/rat, intra-CA1). Two groups of animals received post-training administration of ineffective doses of NMDA (10^−5^ and 10^−4^ μg/rat, intra-CA1).

Four groups of animals received post-training administration of lithium (40 mg/kg, IP) and/or lithium (40 mg/kg, IP) plus quetiapine (2.5 mg/kg, IP) in the presence or absence of NMDA (10^-5^ and 10^-4^ μg/rat, intra-CA1) with 5-min intervals.

***Experiment 5***

This experiment investigated the effects of post-training intra-CA1 microinjection of specific doses of MK-801 (a noncompetitive NMDA receptor antagonist) on the memory improvement induced by quetiapine on the lithium-induced impairment of memory consolidation. Seven groups of animals were used. The control group received post-training microinjection of saline (1 μl/rat, intra-CA1). Two groups of animals received post-training microinjection of ineffective doses of MK-801 (0.0625 and 0.125 μg/rat, intra-CA1).

Another four groups of animals received post-training administration of lithium (40 mg/kg, IP) and/or lithium (40 mg/kg, IP) plus quetiapine (10 mg/kg, IP) in the presence or absence of MK- 801 (0.0625 and 0.125 μg/rat, intra-CA1) with 5-min intervals.

***Verification of cannula placements***

Specimen preparation was conducted as in our previous report (11) as follows: 

Once the tests were completed, the animals were euthanized with carbon dioxide (CO_2_) gas, and 1 μl/rat (0.5 μl on each side, intra-CA1) of 1% methylene blue solution was microinjected to verify the accuracy of the microinjection sites.

Then, the rats were decapitated, and their brains were separated and located in a formaldehyde solution (10%). After ten days, the brains were sliced by a vibroslice device in the transverse plane, and then the microinjection sites were verified according to the atlas of Paxinos and Watson ((34). From the total number of 126 implanted cannulas (intra-CA1), the data from 112 animals with correctly implanted cannulas were included in the statistical analyses.

***Statistical analysis***

For statistical analysis, one-way analysis of variance (ANOVA) for comparison between the effects of different doses of drugs with saline or vehicle was used. If the F-value was significant, a *post hoc* comparison of means was carried out with the Tukey test for evaluating specific group comparisons. In all statistical assessments, *P<0.0*5 was used as the criterion for statistical significance. The data are announced as mean ± standard deviation (SD). Calculations were performed using the SPSS statistical package (SPSS Inc., Chicago, Illinois, USA).

## Results

***Histology***

**Figure 2:**
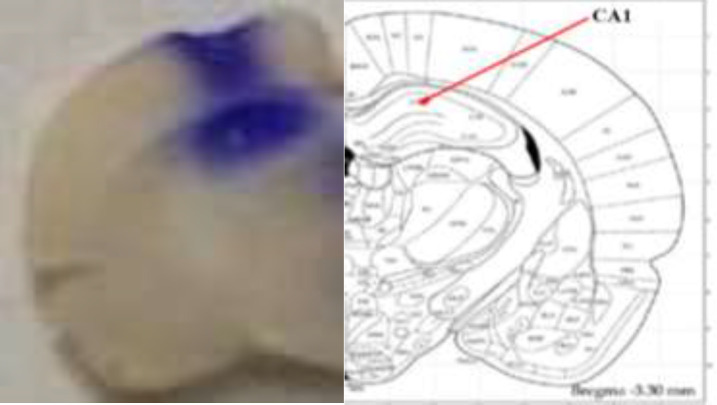
Representative photomicrographs illustrating placement of cannula and needle tip in the CA1 region of the dorsal hippocampus

Figure 2 illustrates the approximate location of the drug microinjections in the CA1 region of the dorsal hippocampus. The histological results were plotted on representative sectors derived from the rat brain atlas by Paxinos and Watson (2007).

***Effects of post-training IP administration of*** ***lithium*** ***on memory consolidation***

**Figure 3:**
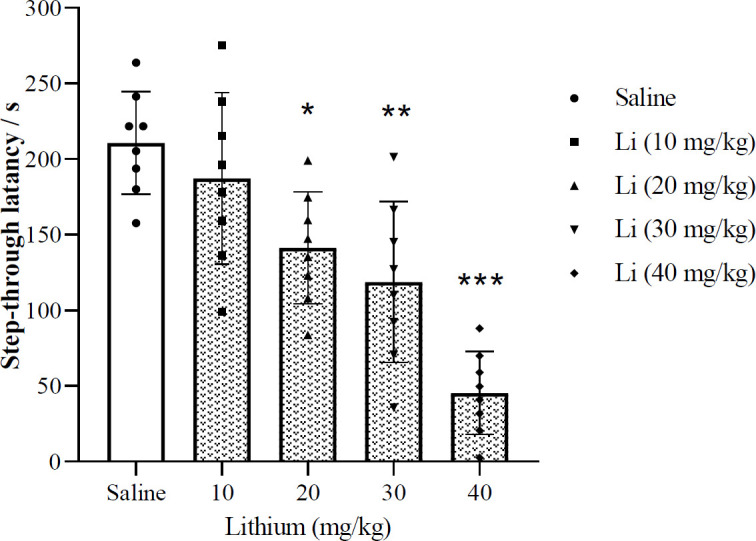
Effects of post-training injection of lithium on memory consolidation

Figure 3 illustrates the effects of post-training injection of different doses of lithium (10, 20, 30, and 40 mg/kg, IP) on memory consolidation. The presented data show that certain doses of lithium (20, 30, and 40 mg/kg) significantly diminished the step-through latency during the retention test (one-way ANOVA; F (4, 35) = 17.35, *P*<0.001). The most significant response was acquired with 40 mg/kg of the drug. Accordingly, the data demonstrate that lithium dose-dependently decreases memory consolidation.

***Effects of post-training IP administration of*** ***quetiapine*** ***on memory consolidation***

**Figure 4:**
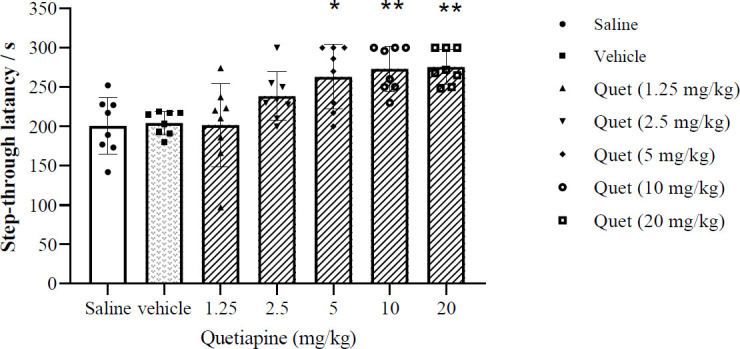
Effect of post-training injection of quetiapine on memory consolidation

Figure 4 illustrates the effects of post-training injection of different doses of quetiapine (1.25, 2.5, 5, 10, and 20 mg/kg, IP) on memory consolidation. The obtained data indicate that the lower doses of quetiapine (1.25 and 2.5 mg/kg) had no significant effect on memory consolidation, while the higher doses of the same drug (5, 10, and 20 mg/kg) significantly augmented the step-through latency during the retention test (one-way ANOVA; F (6, 49) = 7.948,* P*<0.001). The most significant response was acquired with 10 mg/kg of the drug. Accordingly, the data suggest that quetiapine increases memory consolidation.

***Effects of post-training IP administration of*** ***quetiapine on lithium-induced impairment of memory consolidation***

**Figure 5:**
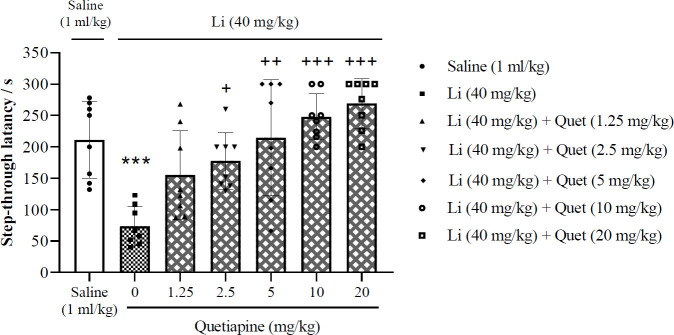
Effects of post-training injection of different doses of quetiapine on lithium-induced impairment of memory consolidation

Figure 5 illustrates the effects of post-training injection of certain doses of quetiapine on lithium-induced impairment of memory consolidation. The presented data displayed that the response induced by lithium (40 mg/kg) significantly was reversed by quetiapine (2.5, 5, 10, and 20 mg/kg) (one-way ANOVA; F (6, 49) = 10.29, *P*<0.001). Hence, the data indicate that quetiapine significantly restored the impairment of memory consolidation induced by lithium.

***Effects of post-training intra-CA1 administration of NMDA on the memory improvement induced by quetiapine on lithium-induced impairment of memory consolidation***

**Figure 6:**
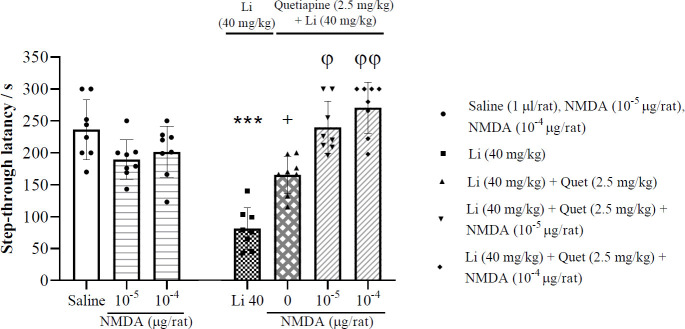
Effects of post-training intra-CA1 microinjection of certain doses of NMDA on the memory improvement induced by quetiapine on lithium-induced impairment of memory consolidation

Figure 6 (left panel) illustrates that the lower doses of NMDA (10^−5^ and 10^−4^ μg/rat) had no significant effect on memory consolidation compared with the saline control group (one-way

ANOVA; F (2, 21) = 2.95, *P*>0.05). As illustrated in the right panel of Figure 6, the memory improvement induced by quetiapine (2.5 mg/kg, IP) on lithium-induced impairment of memory consolidation significantly is potentiated by post-training intra-CA1 microinjection of ineffective doses of NMDA (10^-5^ and 10^-4^ μg/rat) (one-way ANOVA; F (4, 35) = 31.3, *P*<0.01), suggesting the involvement of CA1 NMDA signaling pathway.

***Effects of post-training intra-CA1 administration of MK-801 on the memory improvement induced by quetiapine on lithium-induced impairment of memory consolidation***

**Figure 7:**
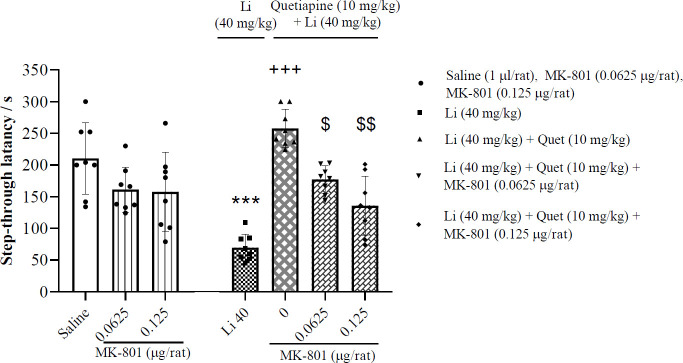
Effects of post-training intra-CA1 microinjection of specific doses of MK-801 on the memory improvement induced by quetiapine on lithium-induced impairment of memory consolidation

Figure 7 (left panel) illustrates that the lower doses of MK-801 (0.0625 and 0.125 μg/rat) had no significant effect on memory consolidation compared to the saline control group (one-way ANOVA; F (2, 21) = 2.54, *P*>0.05). As illustrated in the right panel of Figure 7, the memory improvement induced by quetiapine (10 mg/kg, IP) on lithium-induced impairment of memory consolidation significantly is inhibited by post-training intra-CA1 microinjection of ineffective doses of MK-801 (0.0625 and 0.125 μg/rat) (F (4, 35) = 28.75, *P*<0.01), suggesting the involvement of CA1 NMDA signaling pathway.

The arrow denotes the location of the needle tip and the verified section adapted from the atlas of Paxinos and Watson (Paxinos and Watson, 2007) is also included.

Five groups of animals received a post-training injection of saline (1 ml/kg, IP) and/or lithium (10, 20, 30, and 40 mg/kg, IP). The step-through latency was measured 24 hr after drug injection. Each point is the mean±SD for eight rats.

*P<0.05, **P<0.01, and ***P<0.001 compared with the post-training saline group.

Seven groups of animals received injections of saline (1 ml/kg, IP) or vehicle (1 ml/kg, IP) and/or quetiapine (1.25, 2.5, 5, 10, and 20 mg/kg, IP) immediately after training. The step-through latency was measured 24 hr after drug injection. Each point is the mean±SD for eight rats.

*P<0.05 and **P<0.01 compared with the post-training vehicle group.

Seven groups of animals were injected with saline (1 ml/kg, IP) or lithium (40 mg/kg, IP) immediately after training. Quetiapine (1.25, 2.5, 5, 10, and 20 mg/kg, IP) was injected 5 min after lithium injection. The step-through latency was measured 24 hr after drug administration. Each point is the mean±SD for eight rats.

***P<0.001 compared with the post-training saline group.

+*P*<0.05, ++*P*<0.01, and +++*P*<0.001 compared with the post-training lithium group.

Three groups of animals received intra-CA1 microinjection of saline (1 μl/rat) or specific doses of NMDA (10−5 and 10−4 μg/rat) immediately after training. The other four groups of animals received post-training injections of lithium (40 mg/kg, IP) or lithium (40 mg/kg, IP) plus quetiapine (2.5 mg/kg, IP) in the presence or absence of NMDA. (10−5 and 10−4 μg/rat) with 5-min intervals. The step-through latency was measured 24 hr after drug administration. Each point is the mean±SD for eight rats.

***P<0.001 compared with the post-training saline group.

+*P*<0.05 compared with the post-training lithium group.

φ*P*<0.05 and φφ*P*<0.01 compared with the post-training lithium plus quetiapine (2.5 mg/kg, IP) group.

Three groups of animals received intra-CA1 microinjection of saline (1 μl/rat) or specific doses of MK-801 (0.0625 and 0.125 μg/rat) immediately after training. The other four groups of animals received post-training injections of lithium (40 mg/kg, IP) or lithium (40 mg/kg, IP) plus quetiapine (10 mg/kg, IP) in the presence or absence of MK-801 (0.0625 and 0.125 μg/rat) in 5-min intervals. The step-through latency was measured 24 hr after drug administration. Each point is the mean±SD for eight rats.

***P<0.001 compared with the post-training saline group.

+++*P*<0.001 compared with the post-training lithium group.

$*P*<0.05 and $$*P*<0.01 compared with the post-training lithium plus quetiapine group.

## Discussion

This research aimed to assess the role of dorsal hippocampal (CA1) NMDA glutamate receptors in the interaction effects of lithium and quetiapine on memory consolidation in the step-through inhibitory avoidance task in male rats. 

The present data illustrate that, immediately after training (post-training), IP administration of higher doses of lithium impaired inhibitory avoidance learning memory consolidation. The most significant response was obtained with the 40 mg/kg dose.

These results are in agreement with our prior study and other studies, which have found that systemic and/or intra-CA1 administration of lithium impairs memory formation by altering information coding and synaptic plasticity, resulting in the induction of amnesia in a variety of tasks (9, 11, 12, 35, 36). Hence, the effects of lithium on the brain may be particularly relevant to hippocampal-dependent cognitive processes.

Furthermore, our recent findings indicated that the phosphorylation levels of CAMKII and CREB in the hippocampus and the prefrontal cortex (PFC) are inhibited in lithium-induced memory impairment, suggesting that the hippocampus and the PFC CAMKII-CREB signaling pathway may be involved in lithium’s effect on memory deficits (11). These findings agree with the results of other studies, which reported that acute and/or chronic lithium treatment diminished CREB phosphorylation in the hippocampus and other brain regions (37, 38).

Despite such findings, some researchers have found that lithium had positive effects on memory consolidation using some behavioral tasks (39, 40), which may be due to variations in the amount of lithium administered, duration of drug exposure, site of lithium injection, and the variables examined in different tasks.

Our results also illustrated that post-training IP administration of lower doses of quetiapine (1.25 and 2.5 mg/kg) did not affect memory consolidation, whereas the higher doses of the same drug (5, 10, and 20 mg/kg) improved memory consolidation of inhibitory avoidance learning. In line with such findings, it has been reported that quetiapine enhances memory consolidation and retrieval in a variety of tasks (18, 41). Quetiapine treatment at the lower dose (5 mg/kg) reverses contextual fear conditioning deficits but not spatial reversal deficits in rats treated with kainic acid (an agonist of kainate-class ionotropic glutamate receptors) (42) and improved objective recognition memory in neurodegenerative animal models (15, 33). 

Quetiapine was found to improve the decrease in BDNF-positive cells in the basolateral amygdala and hippocampus of transgenic models of mice with Alzheimer’s disease (43) through its modulating effects on neuroprotective factors such as reducing demyelination and increasing BDNF (44).

Moreover, it could up-regulate the cerebral levels of B-cell lymphoma 2 (Bcl-2) as a neurotrophic factor in Alzheimer’s disease transgenic mice (13, 32).

Despite such findings, it has recently been reported that chronic treatment with quetiapine (25 mg/kg/day for 30 or 90 days) leads to time-dependent impairments in novel object recognition (NOR) performance, enhancements in the pro-BDNF/BDNF ratio, and reductions in Akt and CREB phosphorylation in the hippocampus (45).

This discrepancy may be due to drug doses, acute and/or chronic treatment, type of experiment, and less selective activity on diverse neurotransmitter receptors.

Multiple clinical studies have documented that treatment with quetiapine plus lithium is generally well-tolerated in patients with acute bipolar disorder (BPD) and has greater efficacy than quetiapine alone (46). Interestingly, this study illustrated that specific doses of quetiapine can improve lithium-induced memory impairment (40 mg/kg). These results agree with the findings of other researchers who reported that quetiapine treatment ameliorated reference memory impairment induced by phencyclidine (PCP; an NMDA receptor antagonist) in the radial arm maze task in rats (47, 48). Furthermore, it has been reported that quetiapine improves PCP-induced cognitive deficits in mice in a dose-dependent manner (49).

It has been well documented that the CA1 NMDA receptor signaling pathways have a crucial role in synaptic plasticity, long-term potentiation (LTP), and memory formation (11, 50).

To evaluate whether the CA1 NMDA receptor signaling pathway plays a role in the effects of lithium and quetiapine on memory consolidation, this pathway was activated and/or inhibited by the intra-CA1 administration of NMDA (an NMDA receptor agonist) and MK-801 (a noncompetitive NMDA receptor antagonist), respectively.

Our prior investigations revealed that immediate post-training intra-CA1 microinjection of higher doses of NMDA ameliorates, while MK-801 diminishes memory consolidation using inhibitory avoidance tasks. However, lower doses of the drugs did not affect memory consolidation (22, 30, 51, 52).

Multiple experimental studies have shown that activation of CA1 NMDA receptors is involved in the learning and memory processes in a one-trial inhibitory avoidance task (11, 22). Furthermore, activating CA1 NMDA receptors by its agonists leads to activation of CREB and CaMKII in the CA1 regions of the dorsal hippocampus in rodents using a hippocampal-associated learning task. Although inhibiting CA1 NMDA receptors by their antagonists leads to inhibition of these alterations (11, 53).

In another series of experiments, our findings revealed that intra-CA1 microinjection of the lower doses of NMDA (10^-5^ and 10^-4^ µg/rat), which did not affect memory consolidation by itself, potentiated the memory amelioration induced by co-administration of lithium (40 mg/kg) and quetiapine (2.5 mg/kg), indicating a potentiated effect between quetiapine and NMDA.

These results are in agreement with our prior study and those of other researchers who found that post-training intra-CA1 microinjection of the low dose of NMDA (10^-4^ µg/rat) significantly lessened memory deficit induced by lithium in hippocampal-dependent learning and memory tasks in rodents (11, 12).

Our results also revealed that intra-CA1 microinjection of the lower doses of MK-801 (0.0625 and 0.125 µg/rat), which did not affect memory consolidation by itself, reversed the memory amelioration induced by the co-administration of lithium (40 mg/kg) and quetiapine (10 mg/kg), indicating that the CA1 NMDA receptor signaling pathway may be involved in the interplay among lithium and quetiapine on memory consolidation.

Hence, these findings suggest a functional interaction between quetiapine and lithium via CA1 NMDA receptor mechanisms in inhibitory avoidance learning memory consolidation.

The results are consistent with our previous study and other studies that found that post-training intra-CA1 microinjection of sub-threshold dose of the competitive and noncompetitive NMDA receptor antagonists significantly increases lithium-induced memory deficits using hippocampal-related behavioral tasks (11, 12). In addition, quetiapine has been shown to regulate glutamate receptor activity in the hippocampus and other areas of the brain. The stimulatory effects of quetiapine on monoamines such as norepinephrine, dopamine, and serotonin have been reported to be mediated by NMDA/glutamate receptors (54). Moreover, quetiapine has been shown to reduce schizophrenia-like behaviors, including memory loss, and attenuate BDNF reduction in mice treated with MK-801 (44, 55). Hence, it can be said that quetiapine affects NMDA receptor activity and modulates the effect of lithium on these receptors.

## Conclusion

Overall, the present results indicate that the concomitant administration of lithium and quetiapine could have beneficial effects on memory formation through involvement of NMDA receptors in the CA1 region of the hippocampus. On the other hand, it seems that signaling pathways of CA1 NMDA receptors that modulate synaptic plasticity and memory formation are targets of lithium and quetiapine. Given that memory consolidation is a pivotal component of cognition, the concurrent administration of quetiapine with lithium in BPD therapy is helpful. However, further investigations are needed to elucidate the precise mechanisms involved.

## Authors’ Contributions

 M JS provided concept, methodology, writing the original draft, writing, review, editing, visualization, supervision, project administration, and funding acquisition. S A contributed to formal analysis, investigation, writing the original draft, writing, review, and editing. S E provided formal analysis and investigation. H A and N F contributed to formal analysis, investigation, writing, review, and editing. F K helped with formal analysis, methodology, and writing the original draft.

## Funding

The authors disclosed receipt of the following financial support for the research, authorship, and/or publication of this article: This study was supported by the School of Medicine, Iran University of Medical Sciences, Tehran, Iran (grant number 98-2-4-14898). 

## Conflicts of Interest

 There are no conflicts of interest.
